# Mast cell stabilizing effect of a geranyl acetophenone in dengue virus infection using *in vitro* model of DENV3-induced RBL-2H3 cells

**DOI:** 10.1042/BSR20181273

**Published:** 2019-06-04

**Authors:** Ji Wei Tan, Nuha Fahimah Wan Zahidi, Audrey Siew Foong Kow, Kuan-Meng Soo, Khozirah Shaari, Daud Ahmad Israf, Hui-Yee Chee, Chau Ling Tham

**Affiliations:** 1 Department of Biomedical Science, Faculty of Medicine and Health Sciences, Universiti Putra Malaysia, Serdang 43400, Malaysia; 2 Department of Medical Microbiology and Parasitology, Faculty of Medicine and Health Sciences, Universiti Putra Malaysia, Serdang 43400, Malaysia; 3 Department of Chemistry, Faculty of Science, Universiti Putra Malaysia, Serdang 43400, Malaysia; 4 Current affiliation: School of Science, Monash University Malaysia, Jalan Lagoon Selatan, Bandar Sunway, Subang Jaya 47500, Selangor, Malaysia

**Keywords:** DENV-3, in vitro mast cell degranulation, ketotifen fumarate, RBL-2H3, tHGA

## Abstract

Mast cells (MCs), a type of immune effector cell, have recently become recognized for their ability to cause vascular leakage during dengue virus (DENV) infection. Although MC stabilizers have been reported to attenuate DENV induced infection in animal studies, there are limited *in vitro* studies on the use of MC stabilizers against DENV induced MC degranulation. 2,4,6-trihydroxy-3-geranyl acetophenone (tHGA) has been reported to be a potential MC stabilizer by inhibiting IgE-mediated MC activation in both cellular and animal models. The present study aims to establish an *in vitro* model of DENV3-induced RBL-2H3 cells using ketotifen fumarate as a control drug, as well as to determine the effect of tHGA on the release of MC mediators upon DENV infection. Our results demonstrated that the optimal multiplicities of infection (MOI) were 0.4 × 10^−2^ and 0.8 × 10^−2^ focus forming units (FFU)/cell. Ketotifen fumarate was proven to attenuate DENV3-induced RBL-2H3 cells degranulation in this *in vitro* model. In contrast, tHGA was unable to attenuate the release of both β-hexosaminidase and tumor necrosis factor (TNF)-α. Nonetheless, our study has successfully established an *in vitro* model of DENV3-induced RBL-2H3 cells, which might be useful for the screening of potential MC stabilizers for anti-dengue therapies.

## Introduction

Dengue virus (DENV) infection is an increasing problem in both tropical and subtropical areas and remains endemic in more than 100 countries worldwide. In 2015, the World Health Organization (WHO) reported that an estimated 2.5 million dengue infections occurred globally each year [1,2]. DENV is an arthropod-borne single stranded RNA virus of the *Flavivirus* genus. It is comprised of four distinct serotypes – DENV-1, -2, -3, and -4 – with 65–70% sequence homology and all serotypes contribute to dengue infection in humans [3,4]. DENV infection is presented with a wide range of clinical manifestations, from asymptomatic to mild and self-limiting; to severe and occasionally fatal cases [5]. Typically DENV infection will result in dengue fever (DF), which is a self-resolving febrile disease. Dengue haemorrhagic fever (DHF) and dengue shock syndrome (DSS) are the more severe forms of the infection, and they are characterized by increased vascular permeability and plasma leakage into the tissues [3,6]. Due to limited understanding of the pathogenesis of DENV infection, there is currently no effective therapy or vaccine that is available to treat this disease as well to prevent its transmission [7]. Dengvaxia™, the first approved dengue vaccine, has been used in a few countries but since 2017, its usage in Philippines has been suspended. This is because of its unequal protection against the four different serotypes of the virus [8]. Additionally, persistent protective benefits are seen only in those with prior infection and severe disease could occur following vaccination by seronegative recipients [8]. As such, the search for a new treatment or vaccine against dengue is still needed. Four serotypes of DENV have been found to be co-circulating in Malaysia [9]. However, serotypes are typically area dependent. For instance, DENV1, DENV2, and DENV3 were identified in the state of Negeri Sembilan, whereas multiple entries of DENV2 and DENV4 were reported in the state of Sarawak [10,11]. In populated regions of Kuala Lumpur and Selangor, DENV3 and DENV4 dominated most of the reported DF cases [12]. As such, the DENV used in the present study was type 3 serotype clinically isolated from the hospital located within the region of Selangor, Malaysia.

Recently, the role of mast cells (MCs) has been gaining attention amongst DENV researchers due to their role as a ‘double-edged sword’ in the pathogenesis of DENV infection [13,14]. MC is an important effector cell of the innate immune system, acting as the body’s defence mechanism against any pathogen invasion from surrounding environment including DENV [13]. When activated, MCs immediately release mediators such as histamine and synthesize *de novo* inflammatory mediators, including prostaglandins, leukotrienes, and proinflammatory cytokines [13,15]. Although some studies have suggested a protective role of MCs in the host response against DENV [16], recent studies also suggested that MCs may sometimes have pathogenic role. The release of inflammatory mediators during DENV infection could increase the permeability of capillaries, leading to vascular leakage and subsequently DHF or DSS [14]. Rat basophilic leukemic (RBL-2H3) cell, a type of MC analog, is commonly used to study MC activation. This cell has the ability to release preformed and newly synthesized mediators of immune allergic response following cross-linking of their IgE-bound FcεRI by multivalent allergens [15–18]. RBL-2H3 cell line has been chosen as the cellular model in this preliminary study as there have been earlier studies using this cell line to examine the immune surveillance of MCs during DENV infection [5,19]. In addition, the present study is a continuation of previous studies which reported that RBL-2H3 cells are able to be infected by DENV which, similar to monkey and human MCs, will result in MC activation and degranulation [5,19].

**Figure 1:**
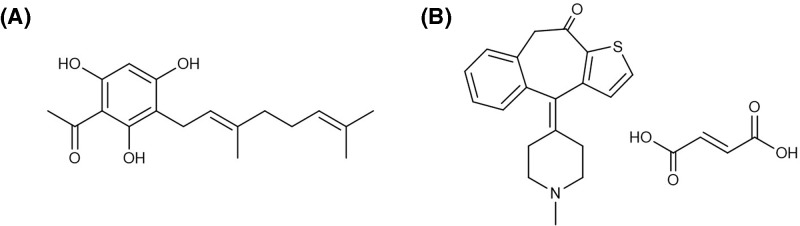
Chemical structures of (A) tHGA and (B) ketotifen fumarate

As MCs have been reported to play a role in the development of DHF and DSS, several new studies have focussed on the use of MC stabilizers as potential treatment against DENV infection. One study successfully demonstrated the use of cromolyn and ketotifen fumarate in reducing vascular leakage in DENV-infected mice [20]. A recent randomized, double-blinded clinical trial study was conducted in Singapore to compare the therapeutic efficacy of DENV-infected patients treated with ketotifen fumarate with those from the placebo group but to date there is no further update from the present study [20]. Although ketotifen fumarate has shown potential in an *in vivo* study, its potential has not been reported in *in vitro* studies. Hence, the first objective of our preliminary study was to establish an *in vitro* model of RBL-2H3 MC degranulation for the screening of potential MC stabilizers in DENV infection using ketotifen fumarate. tHGA is a chemically synthesized active compound originally found in local shrub *Melicope ptelefolia* [21]. This shrub is traditionally used by locals to treat a wide range of diseases. It has also been scientifically reported to exhibit exceptional anti-pyretic, anti-bacterial, analgesic, and anti-inflammatory activities [22]. Recent studies have shown that tHGA may exert MC stabilizing activity by inhibiting IgE-mediated degranulation in RBL-2H3 cells [17,18]. However, there has yet to be a report of the MC stabilizing effect of tHGA in DENV infection. Therefore, the second objective of the present study was to examine the MC stabilizing effect of tHGA in DENV infection using the established *in vitro* model of DENV3-induced RBL-2H3 cells degranulation (Figure 1).

## Results

### Ketotifen fumarate inhibits the release of β-hexosaminidase by DENV3-induced RBL-2H3 cells at MOI(s) of 0.4 × 10^−2^ and 0.8 × 10^−2^ FFU/cell

**Figure 2:**
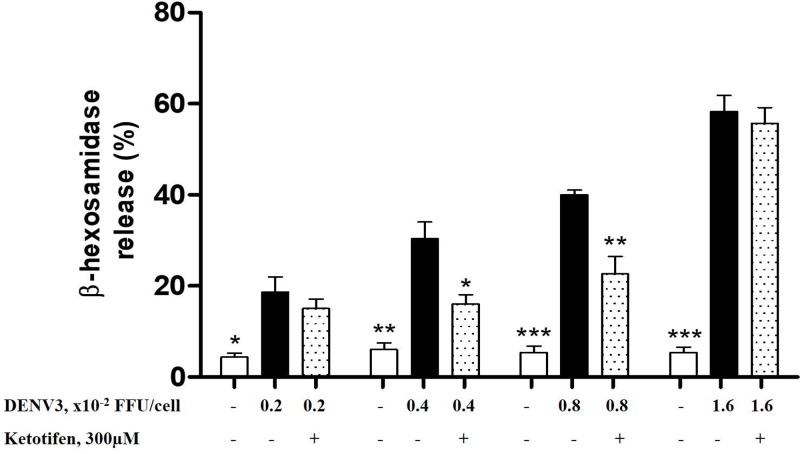
The effect of ketotifen fumarate on the release of β-hexosaminidase by DENV3-induced RBL-2H3 cells using MOIs ranging from 0.2 × 10^−2^ to 1.6 × 10^−2^ FFU/cell RBL-2H3 cells were pretreated with ketotifen fumarate (300 μM) for 20 min followed by DENV3 infection for 1.5 h to induce degranulation. The percentage of β-hexosaminidase released was calculated against the total amount of β-hexosaminidase. Results are expressed as the mean ± S.E.M. of three independent experiments. ***P<0.005, **P<0.01 and *P<0.05 as compared with their respective DENV3-induced RBL-2H3 groups (black bars).

Although the role of β-hexosaminidase in dengue is still unknown, it is an important marker for the quantitation of granule exocytosis during the early stage of MC activation [23]. According to previous study, RBL-2H3 cells infected directly with DENV released β-hexosaminidase [19]. Thus, for better comparison with earlier studies where RBL-2H3 cells were infected by DENV3, the present study measured the degree of degranulation by examining the release of β-hexosaminidase in order to determine the optimal MOI to be used. Per Figure 2, all the MOIs of clinically isolated DENV3 used in the present study were able to significantly induce degranulation in RBL-2H3 cells from 4 to 19% (0.002 FFU/cell), 6 to 30% (0.004 FFU/cell), 5 to 40% (0.008 FFU/cell), and 5 to 58% (0.016 FFU/cell). However, pretreatment of ketotifen fumarate was only able to significantly reduce the release of β-hexosaminidase by 46.5 and 42.9% in the cells infected by 0.004 and 0.008 FFU/cell, respectively. The significant decrease in the release of β-hexosamidase by ketotifen fumarate pretreated RBL-2H3 cells has proven that the use of a MC stabilizer was effective in attenuating DENV infection in a cellular model of MC activation. These findings also indicated that the optimal MOI of clinically isolated DENV3 for the screening of MC stabilizers in DENV infection ranges between 0.4 × 10^−2^ and 0.8 × 10^−2^ FFU/cell. Therefore, this optimized model was used in subsequent experiments to further examine the MC stabilizing effect of tHGA on DENV3-induced RBL-2H3 cells.

### tHGA does not reduce RBL-2H3 cell viability at 20 μM and below

**Figure 3:**
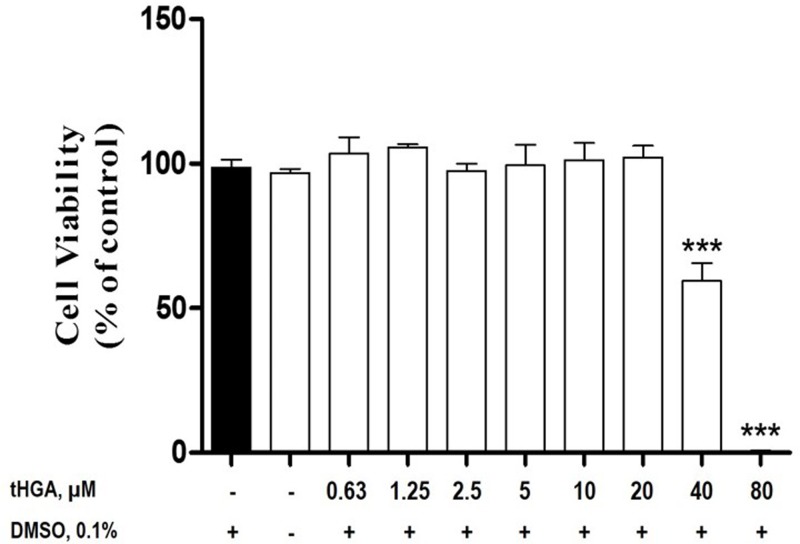
The cytotoxic effects of tHGA on RBL-2H3 cells The RBL-2H3 cells were incubated with increasing concentrations of tHGA (0-80 μM) for 24 h. The cytotoxicity of the treatments was determined by MTT assay. The results are expressed as the mean ± S.E.M values of three independent experiments. ***P<0.005 as compared with the normal RBL-2H3 cells (black bar).

Once the *in vitro* model of DENV3-induced RBL-2H3 cells degranulation has been established using ketotifen fumarate, the present study continued by determining the inhibitory effects of tHGA on the established model. To ensure that the inhibitory effects was not due to the compound’s cytotoxicity on RBL-2H3 cells itself, the cytotoxic effect of tHGA on RBL-2H3 cells was examined using an MTT assay. The results from Figure 3 indicated that tHGA was not cytotoxic to RBL-2H3 cells at the concentrations of 0.63 μM (103.4 ± 5.6%), 1.25 μM (105.7 ± 1.1%), 2.5 μM (97.5 ± 2.5%), 5 μM (99.4 ± 7.2%), 10 μM (101.2 ± 6.0%), and 20 μM (102.2 ± 3.9%). However, tHGA was found to be cytotoxic to RBL-2H3 at 40 μM (59.5 ± 6.1%) and 80 μM (0.5 ± 0.2%). Thus, the three highest non-cytotoxic concentrations of tHGA (1.25, 5, and 20 μM) were used for subsequent experiments in the present study. The drug control ketotifen fumarate is also non-cytotoxic to RBL-2H3 cells at the concentration of 300 μM (data not shown). Similar concentration of ketotifen fumarate has also been used in previous studies related to MC degranulation [17,19,24].

### tHGA does not inhibit the release of β-hexosaminidase by DENV3-induced RBL-2H3 cells

**Figure 4:**
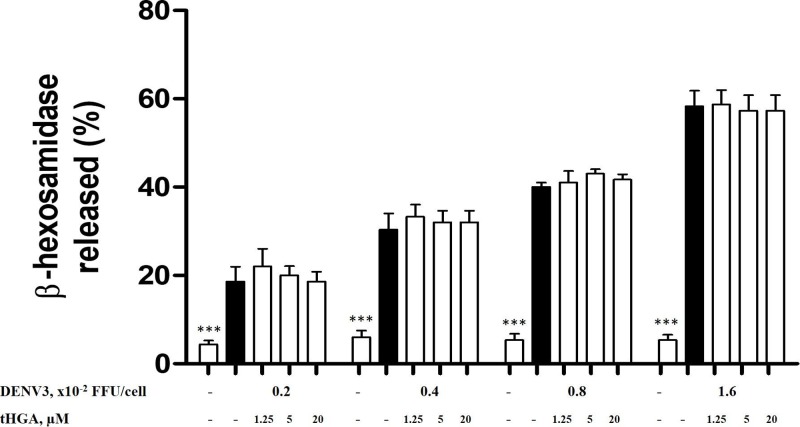
The effect of tHGA on the release of β-hexosaminidase by DENV3-induced RBL-2H3 cells RBL-2H3 cells were pre-treated with tHGA (1.25, 5, and 20 μM) for 20 min followed by the infection of DENV3 at 0.2, 0.4, 0.8, and 1.6 × 10^−2^ FFU/cell for 1.5 h to induce degranulation. The percentage of β-hexosaminidase release was calculated against the total amount of β-hexosaminidase being released by DENV3-induced RBL-2H3 cells. Results are expressed as the mean ± S.E.M. of three independent experiments. ***P<0.005 as compared with the DENV3-induced RBL-2H3 group (black bars).

RBL-2H3 cells were pretreated with the non-cytotoxic concentrations of tHGA (1.25, 5, and 20 μM) or ketotifen fumarate (300 μM) for 20 min and followed by induction with different MOIs of DENV3 for 1.5 h. As per Figure 4, RBL-2H3 cells were able to be induced by all DENV3 MOIs as reflected by the release of β-hexosaminidase - 0.2 × 10^−2^ FFU/cell (19%); 0.4 × 10^−2^ FFU/cell (24%); 0.8 × 10^−2^ FFU/cell (35%), and 1.6 × 10^−2^ FFU/cell (58%). However, tHGA at all concentrations were not able to attenuate the release of β-hexosaminidase.

### tHGA does not inhibit the release of *de novo* mediators by DENV3-induced RBL-2H3 cells

**Figure 5:**
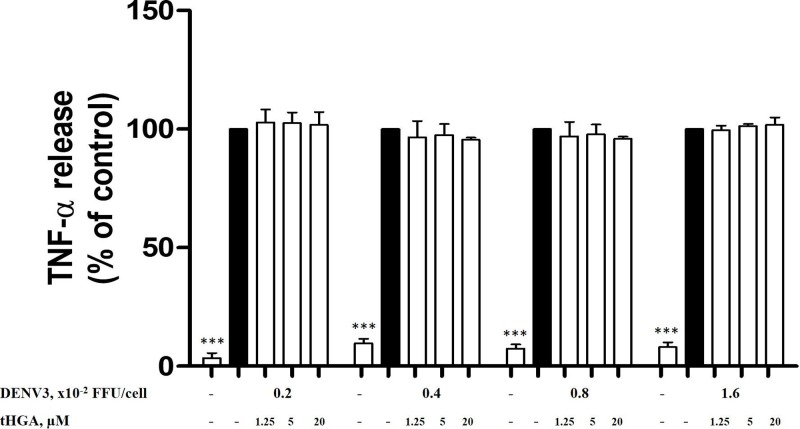
The effect of tHGA on the release of TNF-α by DENV3-induced RBL-2H3 cells RBL-2H3 cells were pretreated with tHGA (1.25, 5, and 20 μM) for 20 min and then infected with DENV3 at 0.2, 0.4, 0.8, and 1.6 × 10^−2^ FFU/cell for 24 h. The level of TNF-α was determined using an ELISA kit by following the manufacturer’s instructions. Results are expressed as the mean ± S.E.M values of three independent experiments. ***P<0.005 as compared with the DENV3-induced RBL-2H3 group (black bars).

Prolonged activation of RBL-2H3 cells by DENV will result in late phase reaction and production of *de novo* inflammatory mediators such as lipid mediators and pro-inflammatory cytokines [25,26]. Even though TNF-α released by MCs is considered as both preformed and *de novo* inflammatory mediator [27], it is still being considered as a worthy mediator to be examined as it has the ability to promote the disruption of cell–cell junction which leads to increased vascular permeability in DHF/DSS [28]. As shown in Figure 5, DENV3 infection for 24 h significantly increased the release of TNF-α in the RBL-2H3 cells. This has confirmed that MCs are responsible for the synthesis and release of *de novo* mediators such as TNF-α in a prolonged period of infection by DENV. However, the level of TNF-α released by DENV3-induced RBL-2H3 cells was not significantly reduced in the presence of tHGA, even at the highest concentration of 20 μM.

## Discussion

With the recent discovery of MC’s involvement in the pathogenesis of DENV infection, researchers have been studying the use of MC stabilizers in treating DF [13,20]. The MC stabilizers are used clinically to prevent allergic reactions to common allergens by inhibiting the release of allergic mediators from MCs [29]. However, information on the use of MC stabilizer against DENV infection remains limited. Cromolyn and ketotifen fumarate have been demonstrated to successfully decrease vascular leakage in activated MC of DENV-infected mice [13]. Another *in vivo* study has shown that ketotifen fumarate was able to reverse many of the responses of DENV infection in mice – reduced metabolic dysregulation, complement signaling and inflammation [30]. As there is yet to be any *in vitro* studies that study the effect of MC stabilizers on DENV-infected MC degranulation, we have in our study introduced a potential *in vitro* DENV-infected MC degranulation model using RBL-2H3 cells. Our results revealed that direct infection of DENV3 was able to induce degranulation in RBL-2H3 cells, and the pretreatment with ketotifen fumarate was able to attenuate the level of β-hexosamidase released by DENV3-induced RBL-2H3 cells. Such results were in line with a previous study, in which RBL-2H3 cells released significant amount of β-hexosamidase as a result of degranulation when infected with DENV (type 1–4) [19]. Interestingly, the degree of degranulation (Figure 2) by RBL-2H3 cells in our study was also comparable with that previous study [19]. The MOI optimization experiment in Figure 2 has shown that ketotifen fumarate was unable to attenuate MCs degranulation when the MOIs were too high (1.6 × 10^−2^ FFU/cell) or too low (0.2 × 10^−2^ FFU/cell). At low MOI, there appeared to be a mild reduction by ketotifen fumarate on the level of β-hexosamidase, but the inhibition was not statistically significant. This may be because low MOI was unable to induce drastic level of β-hexosamidase in comparison with the non-infected cells. At high MOI, the infection could be too strong until the release of β-hexosamidase becomes unable to be inhibited by ketotifen fumarate. Therefore, the MOIs ranging from 0.4 × 10^−2^ FFU/cell to 0.8 × 10^−2^ FFU/cell were used in the subsequent experiments to determine the effect of another potential MC stabilizer, tHGA, in attenuating DENV-3 induced RBL-2H3 cells.

tHGA is a geranyl acetophenone originally found in the young leaves of *Melicope ptelefolia*. It is also a phloroglucinol compound that contains phloroglucinols as its common monomer unit [31]. Previous studies have reported that tHGA, at 5 and 20 μM, exerts anti-allergic properties by preserving the morphology of MCs and attenuating the release of pro-inflammatory mediators in both cellular and animal model of IgE-mediated MC activation whereby inhibition of β-hexosaminidase and TNF-α were reported [18]. However, our results in the present study revealed that this phloroglucinol compound was unable to attenuate the release of both β-hexosamidase and TNF-α inflammatory by the DENV3-induced RBL-2H3 cells. It is possible that tHGA may not target the signaling molecules involved in the mechanism of action (MOA) of direct DENV infection in MCs. As tHGA has been previously reported to target the linker for activation of T cell, which is located along the classical signaling pathway of MC activation [17], our negative results may indicate that the MOA of the direct DENV infection may not involve the classical pathway of MC activation which is activated by the FcεRI receptor. Up to present, the exact MOA of DENV infection in MCs is yet to be demonstrated despite several studies had reported the MOA of DENV infection in other immune cells [32,33]. Amongst which, DENV has been shown to be taken into hepatocytes and endothelial cells by endocytosis through interaction with multiple cell surface receptors [24]. Apart from that, MC also expresses cell surface receptors known as toll-like receptors (TLRs), which upon stimulation produce cytokine and chemokine, cause MC migration and potentially MC degranulation [34]. Our innate immune system is able to detect microorganism invasion and respond toward them through TLR recognition, and a few TLRs have been identified to play a role in recognizing viruses [35]. Specifically, TLR7 and 8 are responsible for the recognition of single-stranded viral RNA [35,36]. As mentioned earlier, DENV is a ssRNA virus; hence, we speculate that the degranulation of RBL-2H3 and the subsequent release of β-hexosaminidase and TNF-α was due to the interaction between the DENV and TLR7 and eight ligands. In this present study, we showed that ketotifen fumarate inhibited the release of mediators from MC during DENV infection. It is possible that the interaction of ketotifen fumarate with TLR7 and 8 or other cell surface receptors has resulted in the inhibition of MC degranulation by interrupting the recognition of TLR7 and 8 with the DENV3. The differential regulatory effects of these MC stabilizers may have resulted from the different MOA in the inhibition of DENV-induced MC activation. This has been further confirmed by a another experiment, which demonstrated that tHGA failed to inhibit MC degranulation induced by calcium ionophore A23187 (Supplementary Data). The artificial stimulation by calcium ionophore A23187, in comparison with the immunologic inducer IgE, was studied to understand whether the effect of tHGA in MCs could be induced by different mechanisms other than cross-bridging of the FcεRI receptors. Differences on the effects of tHGA in MC degranulation in response to stimulation by the two mentioned triggers could be explained by differences in the biochemical pathways involved. tHGA was reported to be a selective inhibitor of LAT in RBL-2H3, and the inhibition of Syk has been considered to be an inhibitory mechanism of degranulation by tHGA. The ability of tHGA to inhibit IgE-stimulated degranulation but not calcium ionophore A23187-stimulated degranulation, suggested that tHGA attenuates MC degranulation via inhibition of upstream signaling molecules associated with the cell surface receptors including LAT and Syk, without blocking the signaling pathways downstream of cytosolic calcium increase.

Nonetheless, due to the heterogeneous nature of MCs (e.g., mucosal vs connective tissue MCs), the negative effects of tHGA against DENV-induced RBL-2H3 cells are only limited in mucosal MCs. Its effect on DENV-infected connective tissue MCs is yet to be determined. Future studies may focus on exploring the MOA of MC stabilizers particularly ketotifen fumarate within the signaling cascade of DENV-induced MC activation and also to confirm if ketotifen fumarate reduces or interferes with the production or infectivity of DENV [3]. It would be interesting to expand the MC repertoire to include human MCs or other MC lines to relate with human dengue disease pathogenesis as the current preliminary study is based on previous reported studies, where the role of MCs and MC stabilizers in DENV infection using rodents were shown. Furthermore, it is important to note that the present study is also a continuation of other previous studies where DENV-induced MC degranulation during primary DENV infection will result in the release of numerous MC products (anti-coagulant heparin, MC-specific proteases, cytokines such as TNF, and other vasoactive factors) that can act on the vascular endothelium and influence the coagulation cascade. These studies have proven that MC activation is already elevated in primary DHF patients and it is further heightened during secondary infection cascade [3,8]. As the main objective of the present study is to examine the direct effect of DENV toward MC degranulation but not to indicate antibody dependent enhancement, antibodies were not incorporated into the experiments performed. However, a previous study by Syenina et al. [5] has already demonstrated that pre-existing IgGs are able to enhance both MC degranulation and MC-dependent vascular leakage during DENV infection.

In conclusion, the present study has successfully established a preliminary *in vitro* model of DENV3-induced RBL-2H3 cells using a clinically approved MC stabilizer, ketotifen fumarate, as the control drug. The present study also showed that tHGA was not able to attenuate DENV3-induced RBL-2H3 cells. Collectively, the findings of the present study suggest that not all MC stabilizers can be potential treatments for DENV infection as this mainly depends on the mode of inhibition of each individual MC stabilizers.

## Materials and methods

### Compound synthesis and preparation

tHGA was synthesized according to a previously described method [37]. The stock solution (20 mM) was prepared according to a previous study [17]. Prior to experiments, the activity of several batches of synthetic tHGA was tested *in vitro* using the β-hexosaminidase release assay. There was minimal variation between different batches of tHGA (<3% variation).

### Reagents

Eagles’ Minimum Essential Medium (EMEM) was purchased from American Type Culture Collection (ATCC) (Manassas, VA, U.S.A.). Fetal Bovine Serum (FBS), antibiotics (penicillin and streptomycin) and trypsin were purchased from Gibco (Rockford, IL, U.S.A.). 4-Nitrophenyl N-acetyl-β-D-glucosaminide (PNAG) was purchased from Sigma Chemical Co. (St. Louis, MO, U.S.A.). The ELISA kit for TNF-α was purchased from R&D systems (Minneapolis, MN, U.S.A.). Ketotifen fumarate was purchased from Santa Cruz Biotechnology (Dallas, TX, U.S.A.). NP-40 surfact-amps detergent was purchased from Thermo Scientific (Waltham, MA, U.S.A.). Carboxymethyl cellulose (CMC) was purchased from Sigma–Aldrich (St. Louis, MO, U.S.A.). Anti-dengue IgG (pan) mouse antibody was purchased from Acris (Herford, Germany). Horse-radish peroxidase (HRP) was purchased from BioLegend (GmbH, Germany), metal enhanced 3′-diaminobenzidine (DAB) peroxidase substrate was purchased from Thermo Scientific (Pierce, Rockford, IL, U.S.A.).

### DENV strain

DENV serotype 3 (DENV-3) was a clinical isolate virus from Hospital Serdang, Selangor, Malaysia. Virus was propagated in *Aedes albopictus* C6/36 mosquito cells (CRL-1660; ATCC) and African green monkey (*Chlorocebus*sp) kidney Vero cells were used in foci forming assay to titer DENV3. Both cells were grown in Eagle’s Minimum Essential Medium (EMEM) (Biowest, South America) with 10% FBS (Biowest, South America).

### Virus quantitation

Foci Forming Assay was performed by seeding 24-well cell culture plate with 2 × 10^5^ cells/ml of Vero cells suspension in each well, one day before the viral infection. After 24 h of incubation, the growth medium was removed when the wells were 70–80% confluent. After washing, all wells were infected with 200 μl of various virus dilutions. The plate was incubated at room temperature (RT) for 1 h for virus adsorption followed by washing to remove any unbound virus particles. The cells were then grown in 2% maintenance media added with 1.5% CMC. After 4 days of incubation at 37°C, the culture medium was discarded and the wells were washed gently with PBS. The cells monolayer was fixed with 300 μl of 4% freshly prepared paraformaldehyde for 30 min at RT. Then, the wells were washed three times with PBS and 300 μl of 1 % NP-40 surfact-amps detergent solution was added to permeabilize the cells for 10 min at RT. The wells were washed three times again with PBS and blocked with 300 μl of 3% skim milk solution prepared in water for 1 h at RT. After washing three times with PBS, the cells were incubated with anti-dengue IgG (pan) mouse antibody diluted 1:500 in 1% skim milk solution, at 37°C for 1 h. After that, the wells were washed three times with PBS and incubated with anti-mouse IgG rabbit antibody conjugated with HRP at 37°C for 1 h at the final concentration of 1:250 in 1% skim milk solution. Finally, 200 μl metal enhanced 3′-diaminobenzidine (DAB) peroxidase substrate was added to each well. After 15 min, the DAB peroxidase substrate was removed and the wells were washed with distilled water for three times. Viral foci were counted under a SMZ 1000 stereomicroscope (Nikon, Tokyo, Japan) and expressed as FFU.

### Cell culture

RBL-2H3 cell line (rat basophilic leukemia cell line) was purchased from the ATCC and cultured in EMEM medium containing 10% FBS, 100 U/ml penicillin and 100 μg/ml streptomycin. RBL-2H3 cells were incubated at 37°C in a 5% CO_2_ humidified incubator and subcultured to new T25 tissue culture flask (3 × 10^5^ cells/flask) or used for assays when cells’ confluency reached 80%. Only RBL-2H3 cells of passage number ranging from 6 to 11 were used throughout the present study.

### Optimization of an *in vitro* model of DENV3-induced MC degranulation for the screening of MC stabilizers in dengue

The MC stabilizer ketotifen fumarate was used as the positive control in the present study. To optimize the optimal MOI of DENV3, the release of β-hexosaminidase upon pre-treatment with ketotifen fumarate was examined as previously described with slight modification [5]. Briefly, RBL-2H3 cells (4 × 10^4^ cells/well) were seeded for 24 h. On the next day, the cells were washed with Tyrode’s buffer before pre-treatment with ketotifen fumarate (300 μM) for 20 min. The pre-treated cells were then directly infected with DENV3 at a MOI ranging from 0.2 × 10^−2^ FFU/cell - 1.6 × 10^−2^ FFU/cell for another 1.5 h (β-hexosaminidase) or 24 h (TNF-α). At the end of the infection period, the measurement of the activity of β-hexosaminidase released from the cells was carried out as previously described [38] with slight modifications. Briefly, the culture supernatant from each group was transferred and centrifuged (17,000 g, 10 min) at 4°C. Triton X-100 solution (1%) was added to lyse the cells to release intracellular β-hexosaminidase. Culture supernatant (50 μl) and cell lysate (50 μl) were transferred into separate wells in a non-tissue culture treated 96-well plate and mixed with 100 μl of substrate solution (1 mM p-nitrophenyl N-acetyl-β-D-glucosamine in 0.08 M citrate buffer, pH 4.5) respectively. After 1 h incubation at 37°C, the reaction was terminated by adding 50 μl of stop solution (0.4 M glycine, pH 10) to each well. Absorbance was recorded at 405 nm with an automated microplate reader (Molecular Devices Versa Max, Sunnyvale, CA, U.S.A.). The percentage of degranulation was calculated via the following formula:

%Degranulation=(OD supernatant)/((OD supernatant+OD Triton X))×100%

On the other hand, to determine the effect of ketotifen fumarate on the release of TNF-α from DENV3-induced RBL-2H3 cells, the collected culture supernatant was centrifuged (17,000 × g, 10 min) at 4°C and the levels of the mediator’s released was measured by using ELISA assay kit respectively by following the manufacturer instructions.

### Cytotoxicity assay

The cytotoxicity levels of various concentrations of tHGA against RBL-2H3 cells were determined using MTT assay as previously described with slight modification [18]. Briefly, RBL-2H3 cells (2 × 10^4^ cells/well) were seeded for 24 h at 37°C in a 5% CO_2_ humidified incubator. On the next day, cells were treated with increasing concentrations of tHGA (0.625–80 μM) for 24 h. The cells were then incubated with 10 μl of 2 mg/ml MTT solution for 4 h and removed prior to the addition of 100 μl of 100% DMSO. Readings were then measured at 570 nm using a microplate reader (Molecular Devices Versa Max, Sunnyvale, CA, U.S.A.).

### Analysis of released β-hexosaminidase and TNF-α

To determine the effect of tHGA on the release of preformed and *de novo* mediators during MC degranulation, the release of β-hexosaminidase upon pre-treatment with tHGA (1.25, 5, and 20 μM) was examined by following the protocol as previously described in the optimization part of the present study using ketotifen fumarate.

### Statistical analysis

All experiments described were performed three times. The results were expressed as means ± S.E.M. Statistical analyses were performed using SPSS 19.0 (Chicago, IL, U.S.A.). One-way analysis of variance (ANOVA) followed by Tukey’s ’est was used to compare the results from different treatment groups. *P* values of less than 0.05 were considered statistically significant.

## Supporting information

**Supplementary Data:** Analysis on the Release of β-hexosaminidase by RBL-2H3 Cells Induced by Calcium Ionophore and Pretreated by tHGA

## Abbreviations

ATCC: American Type Culture Collection
CMC: carboxymethyl cellulose
DENV: dengue virus
DF: dengue fever
DHF: dengue hemorrhagic fever
DSS: dengue shock syndrome
EMEM: Eagles’ minimum essential medium
FBS: fetal bovine serum
FFU: focus forming unit
HRP: Horse-radish peroxidase
MC: mast cell
MOA: mechanism of action
MOI: multiplicities of infection
RBL: rat basophilic leukemic
tHGA: 2,4,6-trihydroxy-3-geranyl acetophenone
TLR: toll-like receptor
TNF: tumor necrosis factor
