# Supplementary material for: Mast cell stabilizing effect of a geranyl acetophenone in dengue virus infection using in vitro model of DENV3-induced RBL-2H3 cells
Source: Biosci Rep. 2019 Jun 4;39(6):BSR20181273. doi: 10.1042/BSR20181273 (PMC6549089; doi:10.1042/BSR20181273)
Supplement: Supplementary file 1 [file bsr20181273_Supp1.pdf]

Supplementary Data

Analysis on the Release of β-hexosaminidase by RBL-2H3 Cells Induced by Calcium Ionophore and Pretreated by tHGA

To determine the effect of tHGA on the release of β-hexosaminidase upon calcium ionophore induction, the following protocol was performed. Briefly, RBL-2H3 cells (4 x 10^4 cells/well) were seeded for 24 hrs at 37°C in a 5% CO_2 humidified incubator. On the next day, cells were treated with different concentrations of tHGA (1.25, 5 and 20 µM) for 20 min. Cells were also treated with positive controls - quercetin (30 µM) for 20 min. After that, the cells were induced with 5 µM of calcium ionophore for 1 hr. Subsequently the release of β-hexosaminidase was determined as in Protocol 4.6 whereby the supernatant of spontaneous released β-hexosaminidase and lysed cells' released β-hexosaminidase were incubated with pNAG for 1 hr and the absorbance readings were read at 450 nm.

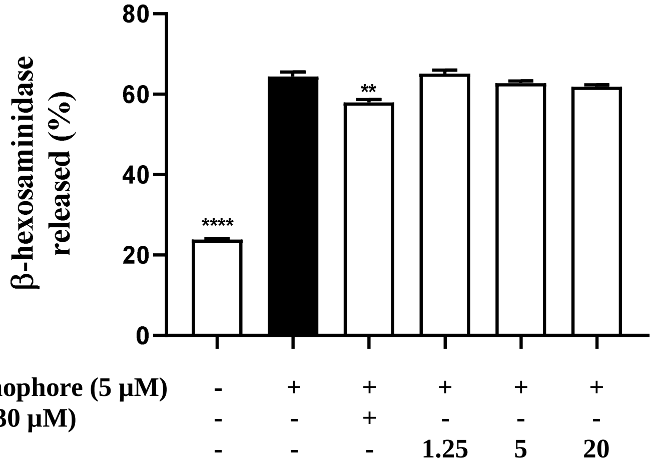

The effect of tHGA on the release of β-hexosaminidase by calcium ionophore-induced RBL-2H3 cells. RBL-2H3 cells were pre-treated with tHGA (1.25, 5 and 20 µM) for 20 min followed by the induction of calcium ionophore at 5 µM for 1 hr to induce degranulation. The percentage of β-hexosaminidase release was calculated against the total amount of β-hexosaminidase being released by calcium ionophore-induced RBL-2H3 cells. Results are expressed as the mean ± S.E.M. of three independent experiments. ****$P<0.005$ as compared to the calcium ionophore-induced RBL-2H3 group (black bar).
